# Characteristics and treatment of congenital perineal groove in male patients

**DOI:** 10.3389/fped.2023.1103867

**Published:** 2023-02-02

**Authors:** Kai Wang, Wenbo Pang, Wei Chen, Dan Zhang, Dongyang Wu, Yajun Chen

**Affiliations:** Department of General Surgery, Beijing Children's Hospital, Capital Medical University, National Center for Children's Health, Beijing, China

**Keywords:** congenital perineal groove, perineal anomaly, anorectal malformation (ARM), characteristics, treatment

## Abstract

**Background:**

Congenital perineal groove (CPG) of male patients has rarely been reported before. The purpose of this study was to review our cases and describe their characteristics and treatment.

**Methods:**

Four male patients diagnosed with CPG were included in this study. Medical records were retrospectively reviewed. Type of CPG and anal position index (API) of the patients were recorded. Follow-up was through outpatient visits.

**Results:**

Their age ranged from 4 years and 2 months to 10 years and 9 months. Among the four patients, two complained of intermittent CPG mucosal hemorrhage and the other two had mucous secreting and soiling. The API was 0.24, 0.35, 0.36, and 0.40 for each patient, respectively, all represented anterior displacement. Type of CPG for the four patients were all partial, and the sulcus was from the posterior perineum to the edge of anus. Two patients were associated with hydrocele, imperforated anus, and rectoperineal fistula; one patient had left varicocele; the remaining patient had sacrum split. All the patients had no postoperative complication, and during the follow-up period of 5–8 months, no symptoms recurred in the four patients; they all had normal defecation.

**Conclusions:**

Both genders share the common three characteristics. In addition, shortened perineum with anterior anus, association of perineal malformations, and partial type occurrence are the extra morphological features in male patients. Furthermore, CPG in males are rarely accompanied by urinary tract infection. Favorable prognosis could be reached after operation.

## Introduction

Congenital perineal groove (CPG) is a rare malformation characterized by the presence of a moist sulcus with mucous membrane on the perineum since birth (1, 2). Because of the rarity, exact incidence cannot be calculated. Articles concerning this topic were mainly case reports and an obvious female predominance was noticed (2, 3). To date, there have only been four male patients documented in the literature (4–7). Thus, characteristics and treatment of male patients have yet to be summarized.

Currently, CPG can be divided into two types based on the morphology of perineum, complete and partial (2, 3, 8). The complete type has the sulcus extended from the posterior fourchette to the anterior edge of the anus in female patients. In the partial type, the sulcus is incomplete and reaches either the posterior fourchette or the anus. This classification can be adopted in male patients when assessing the sulcus from the scrotum to the anus.

The purpose of this study was to retrospectively review our CPG male patients, describe their characteristics and treatment consideration, and discuss the potential pathogenesis.

## Materials and methods

From January 2020 to March 2022, the General Surgery Department of our hospital accepted four male patients who were diagnosed with CPG. The clinical data were retrospectively reviewed with attention to age, manifestations, associated anomalies, and treatment-related information. The CPG type was categorized as complete when the sulcus extended the whole way from the scrotum to the anus, and noted partial if otherwise. Their follow-ups were held through regular outpatient visits.

Anal position index (API) is a quantitative measurement to define the position of anus on the perineum by calculating the ratio of anal–scrotum distance to coccyx–scrotum distance for males, and <0.51 was indicative for anterior displacement (9). Each patient was measured three times, and the mean value was recorded for future analysis.

## Results

**Figure 1:**
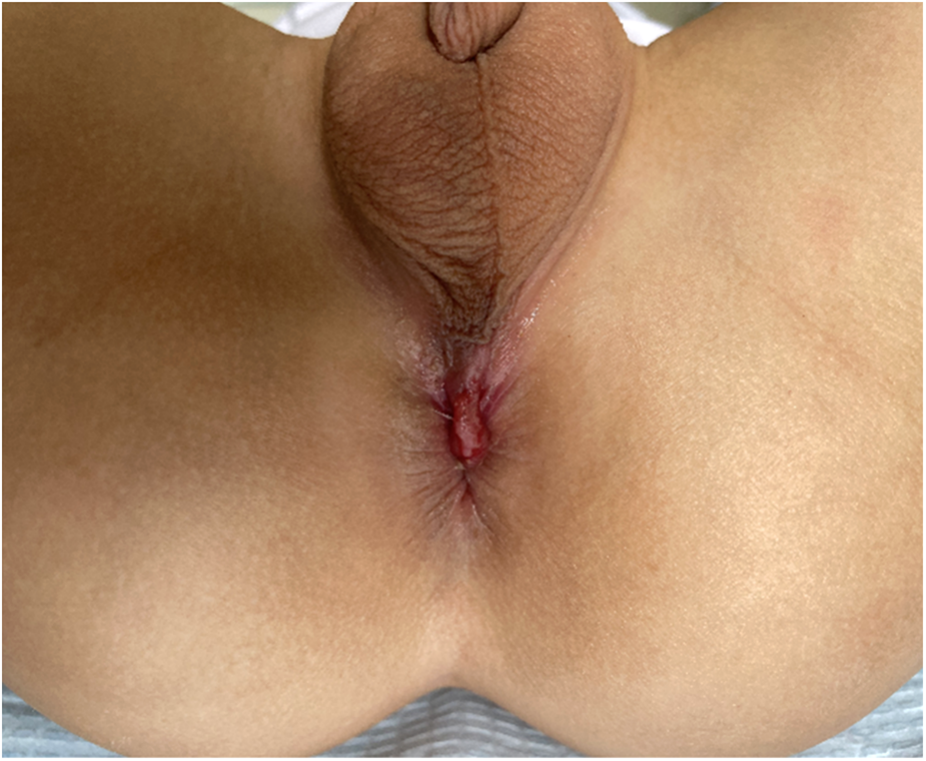
Case 1: Appearance of the perineum showing perineal groove from the posterior perineum to the edge of anus. There was mucous secretion on the sulcus. It was an obvious shortened perineum with an API of 0.24, and the anus was anterior. API, anal position index.

**Figure 2:**
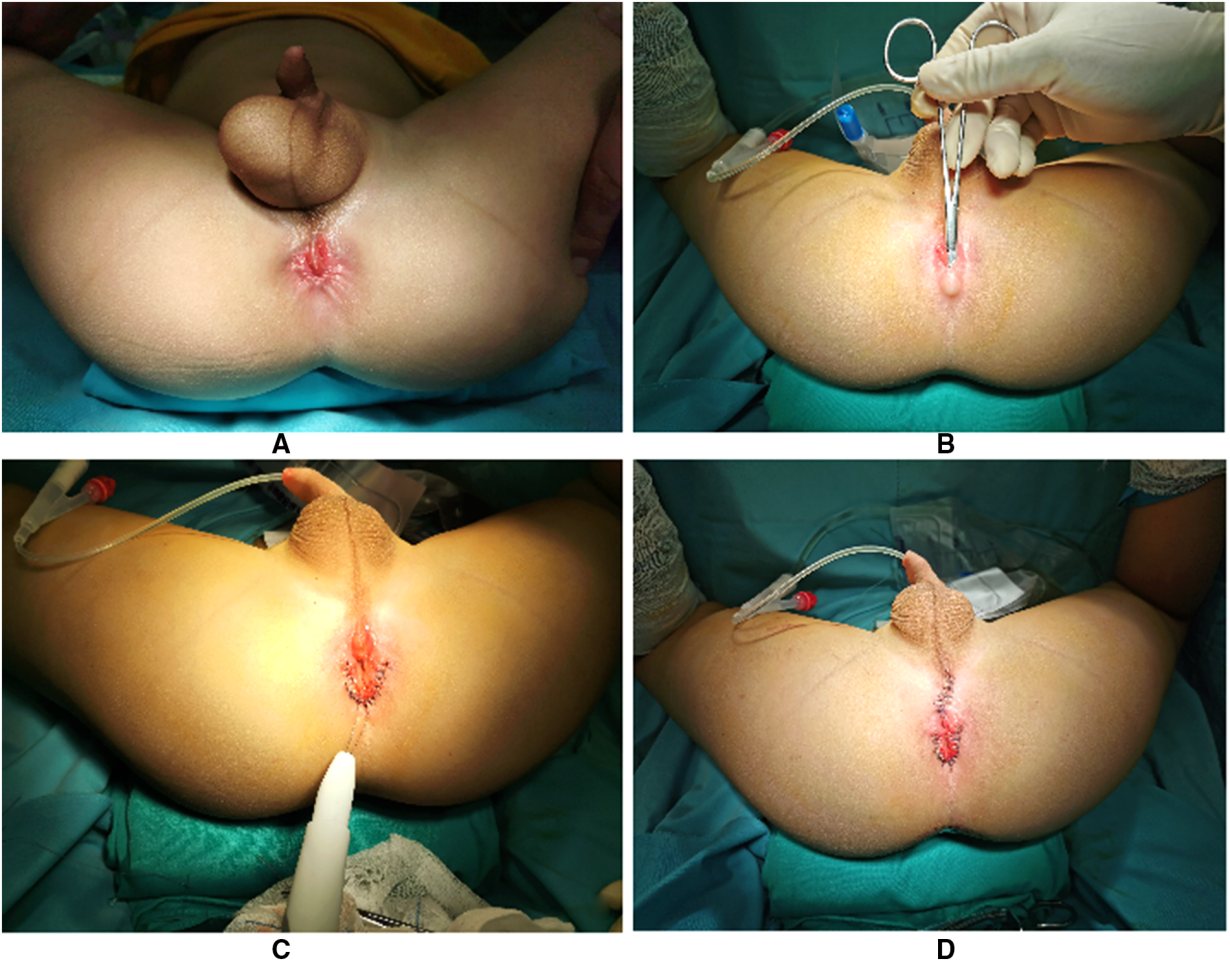
Pictures (**A,B**) of case 2 displaying the right hydrocele, shortened perineum, and imperforated anus with perineal fistula. There was a pouch behind the fistula (**B**). Operation was through cutback anoplasty (**C**) and sulcus mucous membrane removal (**D**).

**Figure 3:**
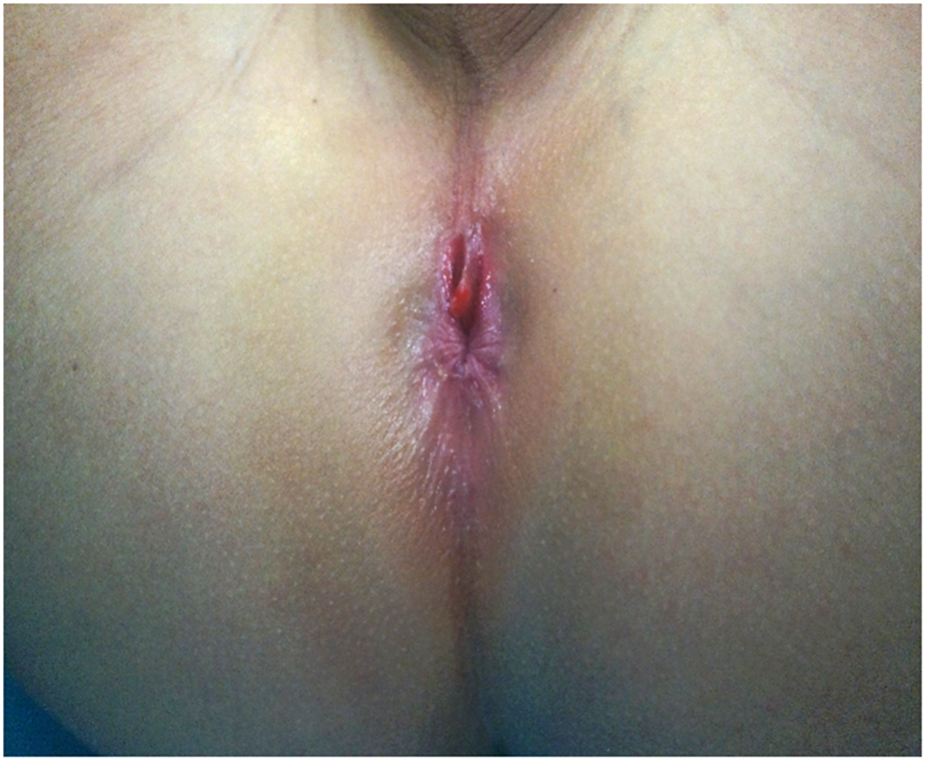
Perineum of case 3 showed a well-delineated, erythematous midline mucous membrane in front of the anus, where there was hemorrhage, accompanied by shortened perineum and anterior anus.

**Figure 4:**
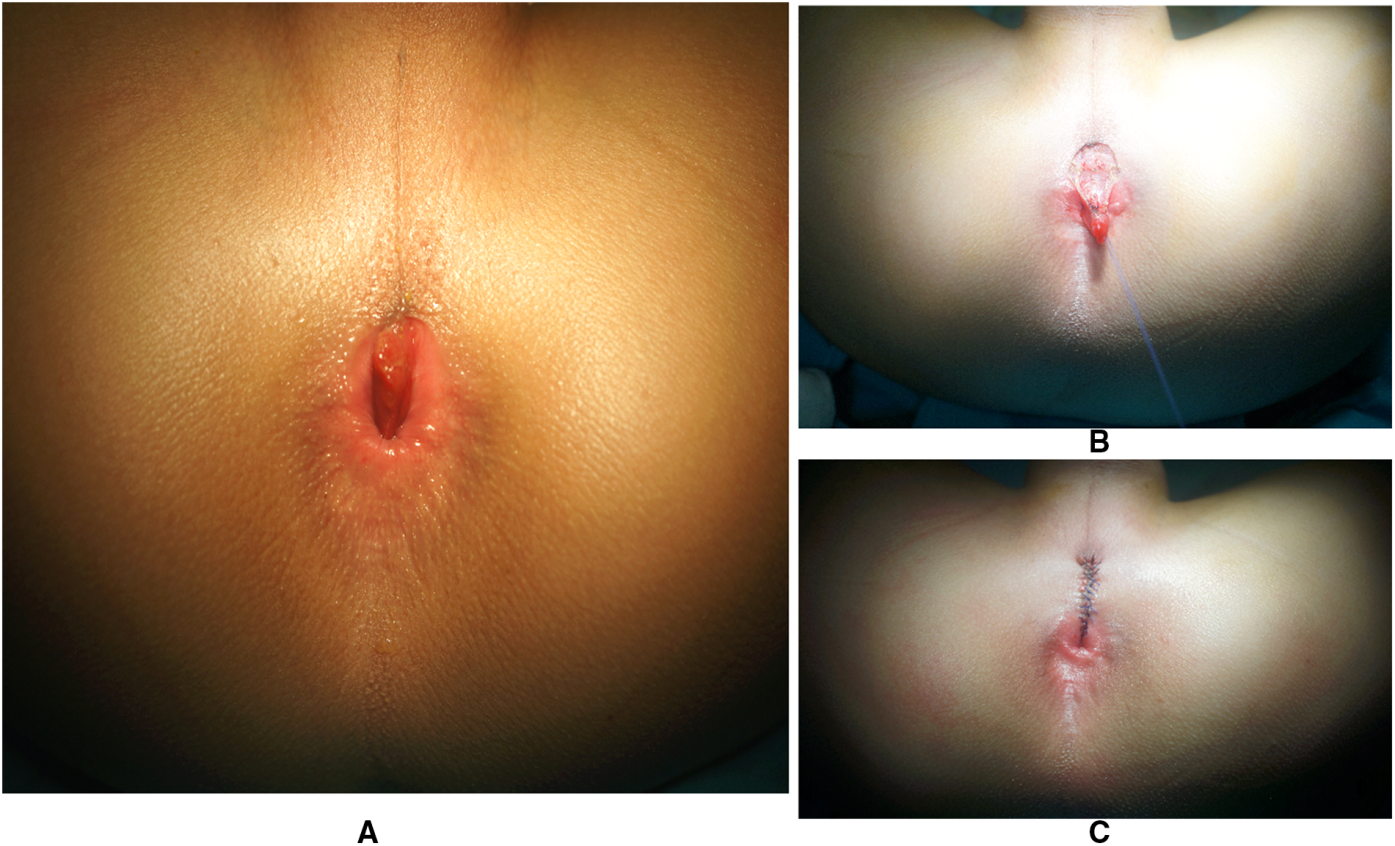
Case 4 had an API of 0.40, and the CPG mucous membrane (**A**) was excised (**B**) with the surrounding tissue anatomical reset and intermittent layered suture *in situ* (**C**). API, anal position index; CPG, congenital perineal groove.

CPG was diagnosed in four male patients in the referring period (Table 1). The age ranged between 4 years and 2 months and 10 years and 9 months. Among the four patients, two complained of intermittent CPG mucosal hemorrhage and the other two had mucous secreting and soiling since birth. No dermatitis, local complication, or urinary tract infection was found in the four patients, and they interacted well with their peers. All four patients had no history of constipation, perineum trauma, or sexual abuse. The API was 0.24, 0.35, 0.36, and 0.40 for each patient, all of which represented anterior displacement, and their perineum appearances were as shown in Figures 1–4, respectively. Types of CPG for the four patients were all partial, and the sulcus was coincident from the posterior perineum to the edge of anus. After analyzing history and comprehensive examinations, one patient had left varicocele; another patient was associated with sacrum split; the remaining two patients had imperforated anus with rectoperineal fistula and hydrocele, and one of them had undergone high ligation of the patent processus vaginalis before. No other anomalies, like hypospadias, penoscrotal transposition, bifid scrotum, or labioscrotal fold deformity, troubled the patients.

**Table 1:** Demographic characteristics of the patients.

Case number	Gender	Age (years)	Symptom	API	Associated malformation	Follow-up time (months)	Prognosis
1	Male	9.50	Mucosal hemorrhage	0.24	Sacrum split	7.50	Good
2	Male	4.22	Mucous secretion	0.35	Imperforated anus with rectoperineal fistula and hydrocele	5.20	Good
3	Male	9.49	Mucosal hemorrhage	0.36	Imperforated anus with rectoperineal fistula and hydrocele	8.50	Good
4	Male	10.82	Mucous secretion	0.4	Varicocele	7.23	Good

API, anal position index.

With the goal of smooth and efficient recovery, all the patients received preoperative intestinal preparation of daily colon irrigation for approximately 7–9 days with diet control. Surgery was through anterior perineal approach (Figures 2, 4) with lithotomy position after general anesthesia. First, we inspected the relationship of the rectum and anal sphincter position by using an electric stimulator to differentiate anterior anus and imperforated anus with rectoperineal fistula (Supplementary Video S1). When the rectum was located in the center of the sphincter, anterior displacement anus was diagnosed (Figure 1), if not it was diagnosed as rectoperineal fistula. In addition, if a pouch could be touched by rectum digital examination, the diagnosis would be rectoperineal fistula (Figure 2B). When a rectoperineal fistula was identified, a cutback anoplasty was done on the first step (Figure 2C). Second, mucous membrane of the sulcus was removed beginning from the perineum to the edge of anus (Figure 4B), continued with anatomical reset and intermittent layered suture *in situ* (Figures 2D, 4C). Urethra was well protected during surgery and catheter was utilized as an indicator for urethra recognition. Pathology of all four patients revealed squamous epithelium.

The patients fasted for the first postoperative week, combined with anus nursing by cleaning and local physiotherapy. They suffered no infection or dehiscence of the wound. From the second week, the two patients who had cutback anoplasty began regular anal dilation for up to 6 months. During a follow-up period of 5–8 months, no symptom recurred in the four patients, and they all had normal defecation.

## Discussion

CPG is a well-delineated, erythematous, non-epithelized mucous membrane on the perineum (10, 11), which was classified as part of the miscellaneous rare group in the 1984 Wingspread classification, and redefined as rare or regional variants in the 2005 Krickenbeck classification (12). Currently, CPG has limited case reports, and most of them are female pediatric patients. After a thorough literature search, information on four male patients was found (4–7), but only two had details. Thus, it is crucial to report the characteristics and treatment of CPG male patients according to our single center experience. Compared with current literature, this study has included the most number of male patients.

Our results showed that the four patients had mucous-related symptoms such as hemorrhage or secretion. Different from female patients, the males had no urinary tract infection. This could be explained by the fact that the urethra and the sulcus has a closer anatomic relationship at the perineum in female patients, especially when the sulcus is the complete type with the posterior fourchette open; mucous secretion would easily contaminate the orifice and induce infection. However, this hardly happens in males as the sulcus is usually the partial type and at a posterior place, separated from the urethra by the scrotums.

In 1968, professor Stephens concluded that female CPG had normal formation of the vestibule, hypertrophy of the minoral tails, and the presence of a wet groove in the perineum between the posterior fourchette and the anus (13). From our study, males may have other extra morphological features. First, API of the four patients all reflected shortened perineum and anterior displacement anus, and two were imperforated anus with rectoperineal fistula. In addition, previous reported cases also had hypospadias, penoscrotal transposition, and bifid scrotum (5, 6). Additionally, all four patients were classified as partial type CPG, and the sulcus was from the posterior perineum to the anus instead of from the scrotum to the anus. Shortened perineum with anterior anus, associated perineal malformations, and the occurrence of partial type CPG may be the morphological characteristics of male patients in addition to the three female features. This can only be further proved by enlarging the sample volume. Explanation for such a phenomenon can be stemmed from the interruption of embryology process of genital folds, labioscrotal folds, and urorectal septum. Normally in the 7th embryo week, development of the urorectal septum separates the cloacal into the anterior urogenital sinus and the posterior anus (13, 14). Then, the labioscrotal folds grow to surround the urogenital sinus bilaterally, and from the 9th week, they begin to descend caudally and fuse at the midline, forming the scrotums in males (10, 13, 15). Meanwhile, the urorectal septum keeps developing, leading to the elongation of the perineum, which pushes the anus backward to its correct anatomical position (15). When various insults interrupt the process and cause defects in extension of the urorectal septum, it would result in a shortened perineum and anterior anus (10). The failure of genital folds fusion at midline would cause sulcus (1, 3, 8, 10, 11, 16). Interruption of labioscrotal fold development contributes to penoscrotal transposition and bifid scrotum (3, 10). Push force from the bilateral mesenchyme promotes the midline fusion (14). Thus, we hypothesized that the forming of scrotums with descending of testis had a larger push force for bilateral fuse than the labia fusion. As a result, it is more rare to encounter CPG male patients, and when diagnosed, they tend to have the partial type of CPG whereby the sulcus ranges from the perineum to the anus instead of scrotum–anus type or scrotum–perineum type. Of course, this hypothesis should be verified by embryological experiments in the future.

CPG is a clinical diagnosis, which is easy to make by inspection (3, 8). It should be differentiated from anal fissure, dermatitis, trauma, or even sexual abuse (3, 8, 11). Apart from the same point of fissure and hemorrhage, patients with anal fissure usually have a definite history of constipation with hard and dry stool, as well as pain defecation that can be relieved when the stool improves. Unlike sulcus, the fissure can be anywhere around the anus in males, unlike the standard 12 o’ clock anal position in CPG. New onset dermatitis can help distinguish from persistent CPG. Trauma or sexual abuse can be ruled out with a clear injury history. Conservation management is recommended for CPG because it is usually asymptomatic and has the potential of self-resolving by epithelialization before the age of 2 years (1, 4, 8, 17). Surgery is only considered when patients are associated with other perineum anomalies that need operation, or over 2 years old with bothersome symptoms (1, 3, 4), like the four patients included in this study. Some patients also received operations due to their parents’ request for cosmetic purposes. Different from females, the urethra should be protected carefully in male patients during surgery, as the sulcus is very close to it. Usage of a urethra catheter is essential before the excision to better indicate and protect the urethra. Postoperative course of the four patients were uneventful, and excellent prognosis was reached, which was in accordance with the literature.

In conclusion, CPG is a kind of regional variant of perineum anomalies, which results from incomplete perineum development. It has a self-resolving potential before the age of 2 years. Unlike females, appearance in male patients is rarer. They share the features of a normal urogenital system formation, hypertrophy of the minoral tails, and wet groove in the perineum. Extra morphological features in male patients include shortened perineum with an anterior anus, association of perineal malformations, and a higher likelihood of being partial type CPG. Furthermore, they are less prone to have a urinary tract infection, and special attention should be paid to the urethra during surgery in case of iatrogenic injury. Favorable prognosis could be reached after operation.

## Data Availability

The original contributions presented in the study are included in the article/**Supplementary Material**, further inquiries can be directed to the corresponding author.
